# A Magnetometer Based on a Spin Wave Interferometer

**DOI:** 10.1038/s41598-017-11881-y

**Published:** 2017-09-14

**Authors:** M. Balynsky, D. Gutierrez, H. Chiang, A. Kozhevnikov, G. Dudko, Y. Filimonov, A. A. Balandin, A. Khitun

**Affiliations:** 1 Department of Electrical and Computer Engineering, University of California -Riverside, Riverside, California 92521 USA; 2 Kotelnikov Institute of Radioengineering and Electronics of the Russian Academy of Sciences, Saratov, 410019 Russia; 3 Saratov State University, Saratov, 410012 Russia

## Abstract

We describe a magnetic field sensor based on a spin wave interferometer. Its sensing element consists of a magnetic cross junction with four micro-antennas fabricated at the edges. Two of these antennas are used for spin wave excitation while two other antennas are used for detection of the inductive voltage produced by the interfering spin waves. Two waves propagating in the orthogonal arms of the cross may accumulate significantly different phase shifts depending on the magnitude and direction of the external magnetic field. This phenomenon is utilized for magnetic field sensing. The sensitivity attains its maximum under the destructive interference condition, where a small change in the external magnetic field results in a drastic increase of the inductive voltage, as well as in the change of the output phase. We report experimental data obtained for a micrometer scale Y_3_Fe_2_(FeO_4_)_3_ cross structure. The change of the inductive voltage near the destructive interference point exceeds 40 dB per 1 Oe. The phase of the output signal exhibits a π-phase shift within 1 Oe. The data are collected at room temperature. Taking into account the low thermal noise in ferrite structures, we estimate that the maximum sensitivity of the spin wave magnetometer may exceed attotesla.

## Introduction

Magnetometers are among the most widely used instruments in a variety of applications^1^. There are different types of magnetic sensors, including superconducting quantum interference devices (SQUID)^2^, resonance magnetometers (e.g. proton magnetometer)^3^, He^4^ e^−^spin magnetometer^4^, solid state magnetometers (e.g. fluxgate, giant magneto-impedance, magneto-resistive, Hall, magneto-electric^5^), and a variety of the fiber optic magnetometers^6–8^. The operation of the above-mentioned magnetometers is based on different physical phenomena offering unique advantages for each type of the magnetic sensors. Sensitivity, intrinsic noise, volume, energy budget, and cost are the most important magnetometer characteristics.

The sensitivity of the magnetic sensor, also referred to as the transfer function, is a characteristic, which relates the input magnetic field to the output voltage ${S}_{B}^{V}(V/T)$ ^9^. The most sensitive sensors show the transfer coefficient as high as 10^5^ V/T^9^. The intrinsic noise of the sensor *B*_*n*_(*f*) is the second important parameter, where *f* is the frequency. The intrinsic noise is usually estimated by measuring the time variation of the output voltage of the sensor followed by the Fourier transform. The result is divided by the transfer function ${S}_{B}^{V}(V/T)$ leading to *B*_*n*,*eq*_(*f*) expressed in *T*√*Hz* ^9^. As of today, SQUID magnetometers demonstrate the highest sensitivity, enabling the detection of extremely subtle magnetic fields, as low as 5 aT (5 × 10^−18^ T) with the noise level of 310^−15^ *T*√*Hz* ^10^. However, the maximum sensitivity of SQUIDs is achieved at the cryogenic temperatures, which translates to a high cost, and narrows down SQUIDs practical applications. In contrast, the solid state magnetometers are compact and capable of operating at room temperature. While less expensive, this type of magnetometers is also less sensitive. The latter motivates the search for highly sensitive solid state sensors operating at room temperature.

One of the promising routes toward highly sensitive solid state magnetometers was proposed in ref. 11. This work demonstrated a prototype device for measuring low alternating magnetic fields by means of ferrite-garnet films with a planar anisotropy. The initial experiments were carried out with Bi-containing RE ferrite-garnet (BiLuPr)_3_(FeGa)_5_O_12_ films, which enablied detection of 10^−7^ Oe magnetic field. More recently, the same group demonstrated a prototype device based on epitaxially grown yttrium iron garnet Y_3_Fe_2_(FeO_4_)_3_ (YIG) films^12^. The 3D YIG magnetometer was experimentally tested, demonstrating the detection level below 10^−12^ *T*√*Hz* at frequencies above 0.1 Hz. The minimum noise level was projected to be at the level of 10^−15^ *T*√*Hz* at room temperature^12^. The high sensitivity of the YIG-based sensor is mainly due to its low intrinsic noise, which makes this material a perfect candidate for magnetic field sensing. In our preceding works, we considered the spin wave propagation and interference in YIG cross-junctions for potential application in memory^13, 14^ and logic devices^15, 16^. The results of our experimental study have revealed prominent interference effect at room temperature. There is an interesting physics of spin wave transport, where the different types of spin waves can propagate and interfere in a cross junction^16^. Spin wave transport in similar T-shaped structures have been studied by other authors as well^17, 18^. In this work, we describe a magnetic field sensor based on the YIG cross-shaped spin wave interferometer.

## Results

### Material Structure and Principles of Operation

**Figure 1:**
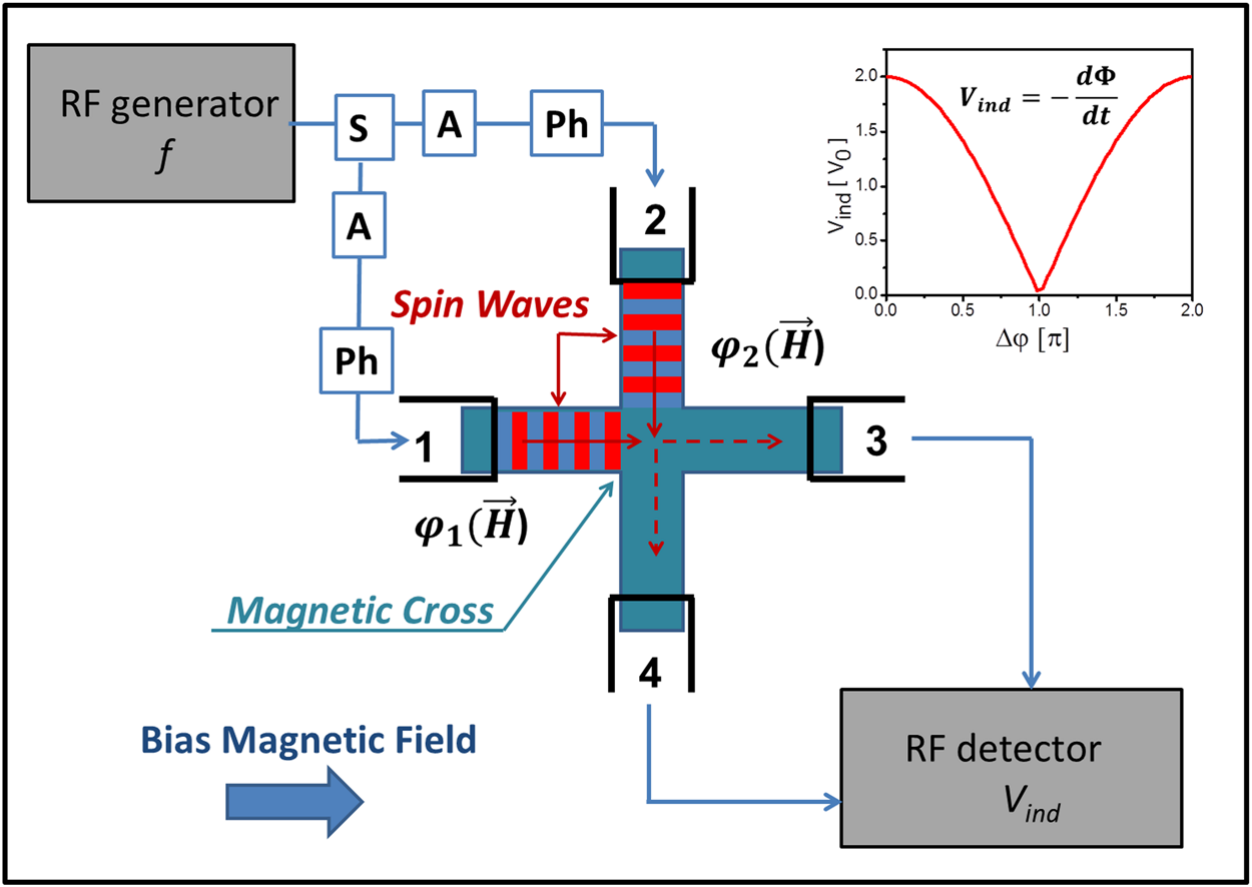
Schematics of the sensing element. It is a spin wave interferometer build on a magnetic cross junction. There are four micro-antennas fabricated on the edges of the cross. Two of these antennas (i.e. ports 1 and 2) are used forthe spin wave excitation, and the other two (i.e. ports 3 and 4) are used for the spin wave detection via the inductive voltage measurements. A sensing element is placed on top of the magnetic substrate, which is aimed to provide a DC bias in-plane magnetic field.

A schematic of the sensor is shown in Fig. 1. The sensing element is a magnetic cross made of a material with the low spin wave damping (e.g. YIG). It is a four terminal device, where the terminals are micro-antennas fabricated on the edges of the cross (e.g. Π-shaped antennas). The antennas are directly placed on top of the cross. Two of these antennas (i.e. marked as 1 and 2) are used for the spin wave excitation while two others (i.e. marked as 3 and 4) are used for the spin wave detection via the inductive voltage measurements^19^. The spin wave generating antennas are connected to the same RF source via the splitter and a set of the phase shifters and attenuators. The output antennas are connected to the detectors. The cross structure is placed on top of the magnetic substrate, which is aimed to provide a DC bias magnetic field (e.g. in-plane magnetic field directed along a virtual line connecting antennas 1 and 3).

The principles of operation of our proposed device can be described in a following way. The input spin waves are excited by passing an RF current through the antennas 1 and 2. AC electric current generates an alternating magnetic field around the current carrying wires, and excites spin waves in the magnetic material beyond the antennas. The details of the spin wave excitation by micro-antennas can be found elsewhere^20, 21^. A spin wave propagates through the cross structure and reaches the output ports. The propagating waves alter the magnetic flux from the structure and induce an inductive voltage *V*_*ind*_ in the output antenna. The output voltage attains its maximum when the spin waves are coming in phase (i.e., constructive interference). The output voltage reaches its minimum when the waves are coming out-of-phase (i.e., destructive interference), as illustrated in the inset to Fig. 1. The phase difference among the waves depends on the external magnetic field *H*, which may produce different phase shifts for the spin waves propagating in the orthogonal arms. Thus, the output voltage depends on the external magnetic field. As we show later, the maximum sensitivity to the magnetic field occurs under the conditions of the destructive interference. In this case, a small phase difference produced by the external field variation results in a significant increase in the output inductive voltage. Below, we describe the physical model of the sensor and estimate its transverse characteristic ∂*V*/∂*H*.

A spin wave is a propagating disturbance of magnetization in ordered magnetic materials^22^, which can be described as a sum of the static ${\overrightarrow{M}}_{0}$ and dynamic $\overrightarrow{m}(r,t)$ components ($|m|\ll |M|$) as follows^23^:

1 $$\overrightarrow{M}(r,t)={\overrightarrow{M}}_{0}+\overrightarrow{m}(r,t).$$

The dynamic component can be described as a propagating wave^24^:

2 $$\overrightarrow{m}(\overrightarrow{r},t)={\overrightarrow{m}}_{0}\cdot exp[-\overrightarrow{\kappa }\overrightarrow{r}]\cdot \exp \,i({\overrightarrow{\kappa }}_{0}\overrightarrow{r}-\omega t+{\phi }_{0}),$$

where ${\overrightarrow{m}}_{0}$ and *φ*_0_ are the initial amplitude and phase, $|\overrightarrow{r}|$ is the distance traveled, *ω* is the frequency *ω* = 2*πf*, and *t* is the time, $\,{\overrightarrow{\kappa }}_{0}$ and $\overrightarrow{\kappa }$ are the real and imaginary parts of spin wave wavevector $\overrightarrow{k}={\overrightarrow{\kappa }}_{0}\,+i\overrightarrow{\kappa }$ ^24^. The $|\overrightarrow{\kappa }|$ is the spatial damping constant, where $|\overrightarrow{\kappa }|\ll |{\overrightarrow{\kappa }}_{0}|$ in the case of low damping waves. The spin waves excited at ports 1 and 2 have the same frequency *ω*, which is defined by the frequency of the input RF signal. The initial amplitudes and phases of the generated spin waves are controlled by the system of phase shifters and attenuators. The generated spin waves propagate through the cross junction and reach the output antennas (e.g., antenna 3 in Fig. 1). The disturbance of magnetization at the output is a result of the spin wave interference:

3 $$\overrightarrow{m}(l,t)={\overrightarrow{m}}_{1}(l,t)+{\overrightarrow{m}}_{2}(l,t),$$

where ${\overrightarrow{m}}_{1}(l,t)$ and ${\overrightarrow{m}}_{2}(l,t)$ are the dynamic components of the spin waves generated at ports 1 and 2, respectively; *l* is the distance traveled. In order to achieve prominent interference effect, we equalize the amplitudes of the spin waves at the output. We use the set of attenuators to compensate possible variation in the input amplitudes $(|\overrightarrow{{m}_{01}}|\ne |\overrightarrow{{m}_{02}}|)$ as well as the difference in spin waves damping $(\lceil \overrightarrow{{\kappa }_{1}}\rceil \ne \lceil \overrightarrow{{\kappa }_{2}}\rceil )$. Hereafter, we assume $|{\overrightarrow{m}}_{1}(l,t)|=|{\overrightarrow{m}}_{2}(l,t)|=\tilde{{m}_{o}}$. In this case, the amplitude of the magnetization change, caused by the spin wave interference, can be expressed as follows:

4 $$m(l,t)=\tilde{{m}_{0}}\cdot \sqrt{2+2\,\cos \,{\rm{\Delta }}\phi }\cdot \,\sin (\omega t+\theta ),$$

where Δ*φ* is the phase difference between the interfering waves, *θ* = atan(sinΔ*φ*/(1 + *cos*Δ*φ*)). The phase difference Δ*φ* is a sum of two parts:

5 $${\rm{\Delta }}\phi ={\rm{\Delta }}{\phi }_{0}+{\rm{\Delta }}\phi (H),$$

where Δ*φ*_0_ is the difference in the initial phases, and Δ*φ*(*H*) = *φ*_1_(*H*) − *φ*_2_(*H*) is the phase difference, which arises during the spin wave propagation. The phase shift accumulated by *i-th* (*i* = 1, 2) spin wave during the propagation is given by

6 $${\phi }_{i}(H)={\int }_{0}^{l}\overrightarrow{{k}_{i}}(\overrightarrow{r})d\overrightarrow{r},$$

where the particular form of the wavevector $\overrightarrow{k}(\overrightarrow{r})$ dependence varies for magnetic materials, film dimensions, the mutual direction of wave propagation and the external magnetic field^25^. For example, the spin waves propagating perpendicular to the external magnetic field (magnetostatic surface spin wave – MSSW) and the spin waves propagating parallel to the direction of the external field (backward volume magnetostatic spin wave – BVMSW) may obtain significantly different phase shifts for the same field. The ratio of the phase shift *Δϕ* to the external magnetic field variation *δH* in the ferromagnetic film can be expressed as follows^26^:

7 $$\begin{array}{lll}\frac{{\rm{\Delta }}\phi }{\partial H}=\frac{l}{d}\,\frac{{(\gamma H)}^{2}+{\omega }^{2}}{2\pi {\gamma }^{2}{M}_{S}{H}^{2}}, & k\parallel H & ({\rm{BVMSW}})\\ \frac{{\rm{\Delta }}\phi }{\partial H}=\frac{l}{d}\,\frac{{\gamma }^{2}(H+2\pi {M}_{S})}{{\omega }^{2}-{\gamma }^{2}{(H+2\pi {M}_{S})}^{2}}, & k\perp H & ({\rm{MSSW}})\end{array}$$

where Δ*φ* is the phase shift produced by the change of the external magnetic field *δH*, *d* is the thickness of the waveguide, *γ* is the gyromagnetic ratio, *4πM*_*s*_ is the saturation magnetization of the magnetic material. The formula above are derived for an approximate dispersion law and valid for *δH* ≪ *H* ^27^.

Propagating spin waves alters the magnetic flux Φ_*m*_ from the structure, which results in the inductive voltage *V*_*ind*_ according to the Faraday’s law of induction:

8 $${V}_{ind}(t)=-\frac{d{{\rm{\Phi }}}_{m}}{dt}=\gamma \frac{dm(l,t)}{dt},$$

where γ is a constant parameter, which accounts for the geometry and material properties of the antenna (e.g. the area and the shape of the antenna contour, antenna’s resistance)^19^. The average output voltage can be found as:

9 $${\bar{V}}_{ind}={\bar{V}}_{0}\cdot \sqrt{2+2\,\cos \,{\rm{\Delta }}\phi },$$

where ${\bar{V}}_{0}$ is the average inductive voltage produced by just one spin wave generating antenna. The set of eqs (1–9) connects the output inductive voltage to the phase difference among the interfering spin waves, which, in turn, relates it to the external magnetic field.

To find the regions of parameters providing maximum sensitivity, we present the results of numerical modeling. The response characteristic of the proposed sensor ∂*V*/∂*H* is defined by the two major factors: (i) spin wave phase sensitivity to the external magnetic field, and (ii) minimum phase shift which can be detected via the inductive voltage measurements:

10 $$\frac{\partial V}{\partial H}=\frac{\partial V}{\partial ({\rm{\Delta }}\phi )}\,\cdot \,\frac{\partial ({\rm{\Delta }}\phi )}{\partial H}.$$

**Figure 2:**
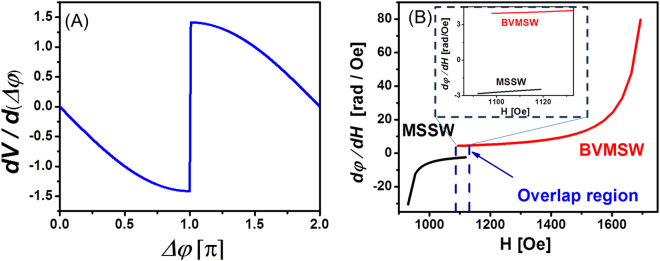
Results of numerical simulations. (**A**) Output inductive voltage as a function of the phase difference between the two interfering spin waves. (**B**) Phase sensitivity of the propagating spin waves to the external magnetic field variation. The material parameters are the following. 4πM_s_ = 1750Gs, γ = 2π·2.80 MHz/Oe; *l/d* = 960. The black and the red curves show the phase sensitivity of BVSM and MSSW types of waves, respectively.

In Fig. 2(A), we present numerical data showing the change of the output inductive voltage as a function of the phase difference between the two interfering spin waves ∂*V*/∂(Δ*φ*). According to eq. (8), the maximum change of the inductive voltage occurs in the case of the destructive wave interference Δ*φ* = *π*, where a small change of the phase difference results in a drastic increase of the inductive voltage.

In Fig. 2(B), we present results of the numerical modeling showing the phase change accumulated by the propagating spin wave due to the magnetic field variation ∂*φ*/∂*H* according to eq. (7). The material parameters used in numerical simulations are the following: 4πM_s_ = 1750 Gs; γ = 2π · 2.80 MHz/Oe; *l/d* = 960; *f* = 4.95 GHz. The black and the red curves in Fig. 2(B) show the phase sensitivity of the MSSW and BVMSW type of the waves, respectively. According to the results of numerical modeling, the sensitivity of the MSSW phase to the external field variation waves increases with the decrease of the bias magnetic field *H*, while the sensitivity of BVMSW phase to the external field variation increases with the increase of the bias magnetic field *H*. Moreover, there is only a finite frequency overlap, where both types of the spin waves can propagate. The overlap occurs due to the effect of the shape anisotropy in the cross junction^16^. For the chosen material parameters and operational frequency *f* = 4.95 GHz, the overlap takes place around H = 1100 Oe, as shown in Fig. 2(B). The width of the overlap is about 30 Oe. The inset in Fig. 2(B) demonstrates the phase sensitivity ∂*φ*/∂*H* of MSSW and BVMSW type of waves: ∂*φ*/∂*H* < 0 for MSSW, and ∂*φ*/∂*H* > 0 for BVMSW. This asymmetry in the phase change is important for the magnetometer functionality. As we discuss in the next section, the proposed magnetometer allows one to detect not only the change in the magnitude of magnetic field but also in its direction.

### Numerical Modeling

**Figure 3:**
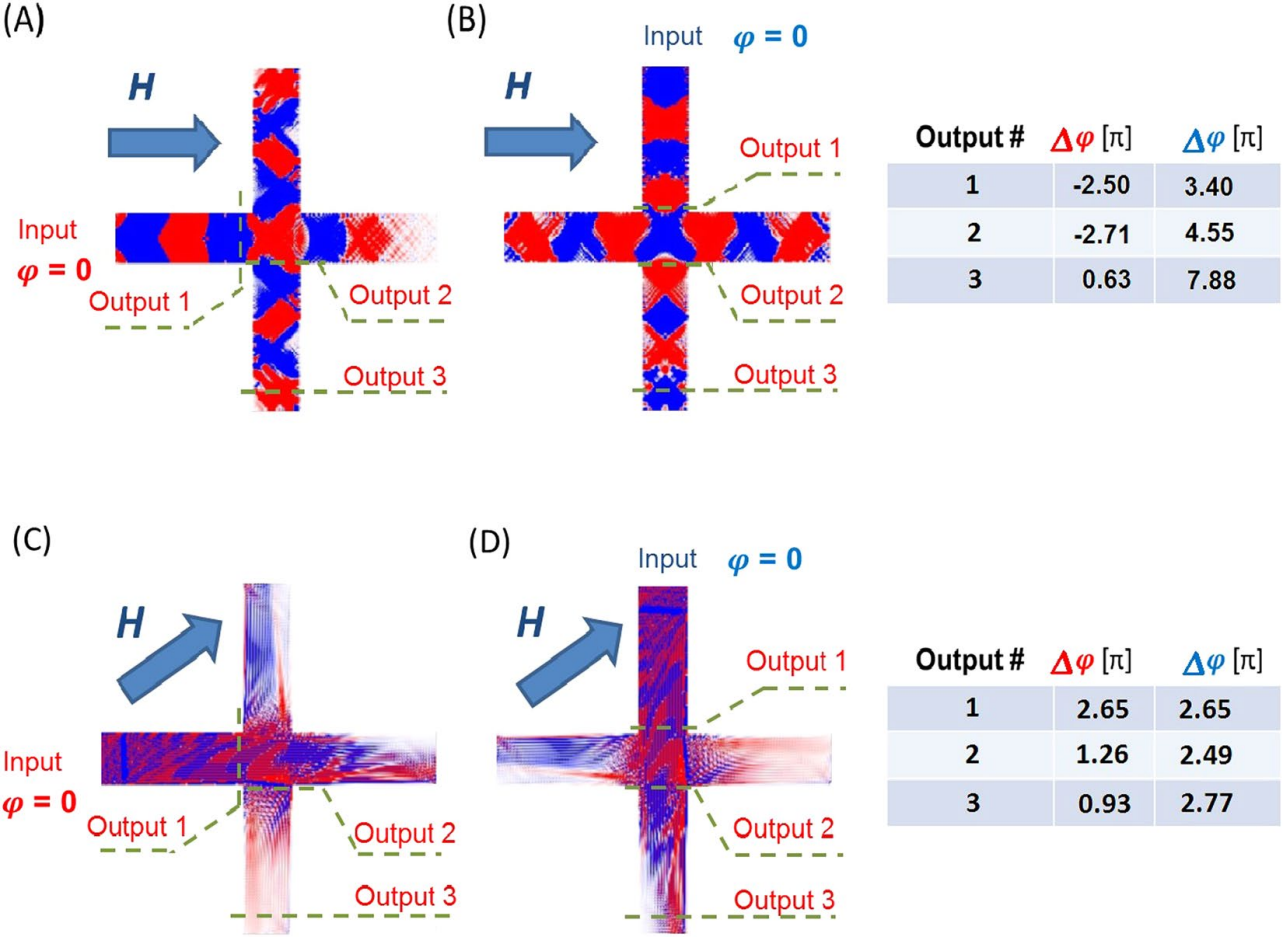
Results of micromagnetic simulations. The color graphs show the snapshots for time t = 100 ns of the out-of-plane component of magnetization. The red and the blue color depict the maxima and the minima of the magnetization projection. (**A**) Bias magnetic field is directed along the virtual line connecting ports 1 and 3. Input spin wave is generated at port 1. (**B**) Bias magnetic field is directed along the virtual line connecting ports 1 and 3. Input spin wave is generated at port 2. The Table (**A**,**B**) on the right shows the phases of the propagating spin waves detected at the three chosen outputs. (**C**) Bias magnetic field is directed at 45^0^ to the virtual line connecting ports 1 and 3. Input spin wave is generated at port 1. (**D**) Bias magnetic field is directed at 45^0^ to the virtual line connecting ports 1 and 3. Input spin wave is generated at port 2. The Table (**C**,**D**) on the right shows the phases of the propagating spin waves detected at the three chosen outputs.

In order to illustrate the process of phase difference accumulation, we present the results of numerical modeling using OOMMF^28^. The test structure is a cross with the following dimensions. The length of the arm is 3.5 mm, the width of the arm is 0.5 mm, and the thickness is 4 μм. The material parameters are: M_s_ = 139 × 10^3^ A/m; the exchange coupling A = 6.5 × 10^−12^ J/m; the damping *α = *0.001. There is a bias in-plain magnetic field Н = 1 kOe. The frequency of the input signal is 4.64 GHz. The elementary cell is a parallelepiped with the dimensions 10 μм × 10 μм × 4 μм. The time step is 10 ps. In Fig. 3, we present a collection of simulated snapshots of the out-of-plane component of magnetization, where the red and the blue color depict the maxima and the minima of the magnetization projection, respectively. Figure 3(A) and (B) correspond to the case when the bias magnetic field is applied along the one of the arms (e.g. virtual line connecting ports 1 and 3). Figure 3(C) and (D) correspond to the case when the bias magnetic field is applied at 45^0^ to the arms. Each snapshot is taken at the time t = 100 ns. We choose 3 control sections on the cross junction depicted as Output 1, Output 2, and Output 3, to trace the phase of the propagating spin wave signal. Output 1 is located just before the cross center. Output 2 is located just after the cross center. Output 3 is located at the output port. The phase at each output is obtained by summation over the cells located beyond the control line (i.e. the dashed green line).

In Fig. 3(A), we present results corresponding to the case when the input wave is generated at port 1 as a BVMSW. The wave accumulates a phase shift about −2.5π with respect to the input while propagating through the arm towards the center of the cross. An additional phase shift of −0.21π is gained during the spin wave propagation through the cross junction. The phase difference between the Output 2 and the Input is −2.71 π. Finally, the wave comes to the Output 3 as MSSW gaining a positive phase shift of +3.34 π. The total accumulated phase (i.e., the phase difference between the Output 3 and the Input) is 0.63π. The Table on the right in Fig. 3 show the accumulated phase subtracted to the odd number of π. Figure 3(B) shows results obtained for the case where the input signal is generated at port 2 as MSSW. The relative phases at the Outputs 1–3 are 3.4 π, 4.55 π, and 7.88 π, respectively. As one can see from Fig. 3(A) and (B), there is a phase difference (Δφ > 15/2 π) between the spin waves coming to the output port (i.e., Output 3). The difference arises due to the two factors: (i) difference in the dispersion (e.g., MSSW and BVMSW waves in the orthogonal arms), and (ii) difference accumulated in the center of the cross (i.e., one wave propagates strait through, the other wave follows a 90^0^ turn). Comparing the phases at Outputs 1 and 2, one can understand the relative contribution of each of them. In the case of magnetic field applied along one of the cross arms, the total phase difference between the two spin waves is defined by the phase accumulated in the cross arms and in the cross center.

There is a special case where the bias magnetic field is applied at 45^0^ to the cross arms as shown in Fig. 3(C) and (D). In this scenario, the waves propagating through the cross arms possess the same dispersion due to the structure symmetry. The results of numerical modeling show zero phase difference between the waves just before the cross center (i.e. Output 1). However, the difference occurs while the waves propagate through the cross center as one of the wave propagates straightforward and the second wave follows a 90^0^ turn. It is expected that the 45^0^ configuration will have the lowest sensitivity to the bias magnetic field variation as the phase difference among the interfering spin waves occurs only in the center of the cross. One should note that the specific details of the spin wave propagation in magnetic cross junctions are still subjects of debates, which require further investigation.

### Experimental Data

**Figure 4:**
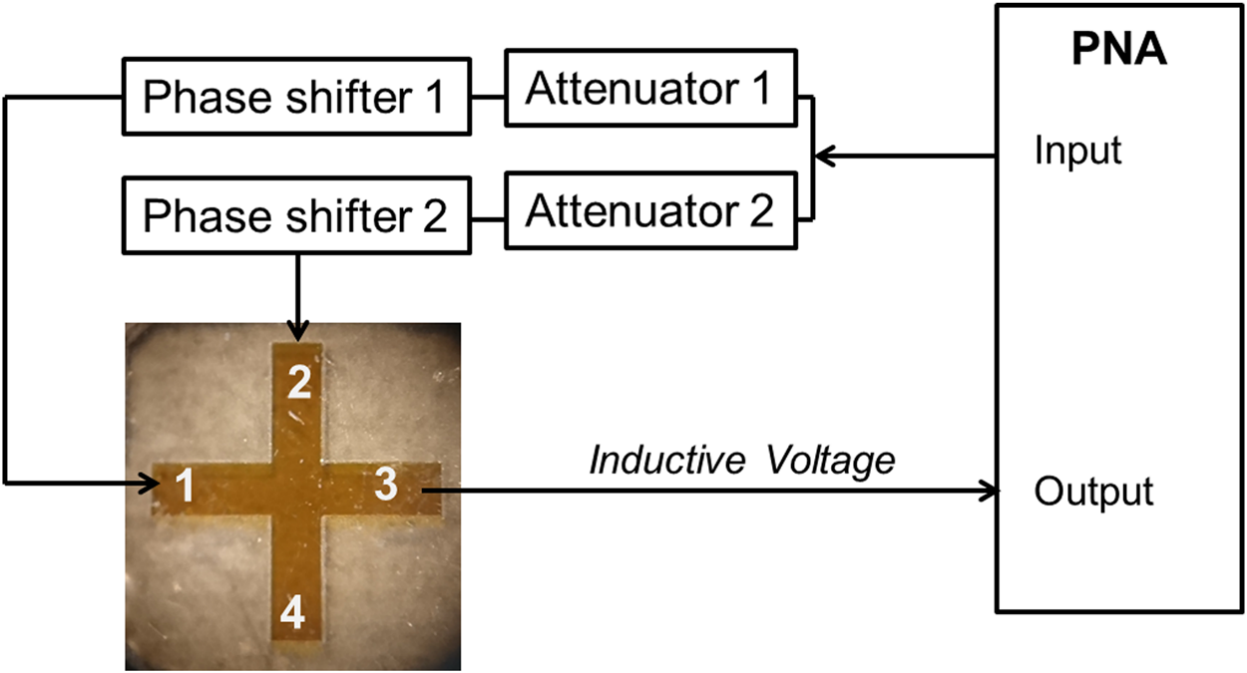
Schematics of the experimental setup. The sensing element is a cross junction made of single crystal Y_3_Fe_2_(FeO_4_)_3_ film. The YIG cross has the following dimension: the length of the each waveguide is 3.65 mm; the width is 650 µm; and the YIG film thickness is 3.8 µm. There are four Π-shaped micro-antennas fabricated directly on the surface of YIG at the edges of the cross. The antennas are connected to a programmable network analyzer (PNA) Keysight N5241A. There is a set of attenuators (PE7087) and a phase shifters (ARRA 9428A) to independently control input power and the phase of the spin wave signals generated at the input ports 1 and 2. The device is placed inside an electromagnet to control the in-plane bias magnetic field from −1000 Oe to +1000 Oe.

A photographic image of the sensing element and connection schematics are shown in Fig. 4. The element is a cross junction made of a single crystal YIG film. The film was grown on top of a gadolinium gallium garnett (Gd_3_Ga_5_O_12_) substrate using the liquid-phase epitaxy technique. The micro-patterning was performed by the laser ablation using a pulsed infrared laser (λ ≈ 1.03 μm), with a pulse duration of ~256 ns. The YIG cross has the following dimensions: the length of the each waveguide is 3.65 mm; the width is 650 µm; and the YIG film thickness is 3.8 µm; and saturation magnetization of 4*πM*_0_ ≈ 1750 *Oe*. There are four Π-shaped micro-antennas fabricated on the edges of the cross. The antennas were fabricated from a gold wire of thickness 24.5 µm and placed directly at the top of the YIG surface. The antennas are connected to a programmable network analyzer (PNA) Keysight N5241A. Two of the antennas marked as 1 and 2 in Fig. 4 are used to generate two input spin waves. The inductive voltage is detected by only one antenna marked 3 in Fig. 4. The details of the inductive measurement technique can be found elsewhere^29^. There is a set of attenuators (PE7087) and a phase shifters (ARRA 9428 A) to independently control input power and the phase of the spin wave signals generated at the input ports 1 and 2. The device was placed inside an electromagnet GMW model 3472–70, pole cap 50 mm (2 inch) diameter tapered, which provides uniform bias magnetic field *ΔH/H* < 10^−4^ per 1 mm in the range from −2000 Oe to +2000 Oe. The electromagnet is needed for the test experiments aimed to identify the most robust regions of operation. Before the experiment, we determined the region in the frequency-bias magnetic field space where both types of waves BVMSW and MSSW can propagate as described in ref. 16. The most prominent overlap takes place in the frequency range from 4 GHz to 5 GHz and bias magnetic field from 750 Oe to 1200 Oe.

The experimental procedure includes two major steps. First, we use the system of attenuators and phase shifters to ensure the destructive spin wave interference at the output port 3. The amplitudes of the spin waves coming to the output port are equalized by the attenuators. Then, we measure the output voltage for the phase difference between the interfering spin waves from 0π to 2π, where the phase difference is controlled by the phase shifters. The minimum of the inductive voltage corresponds to the destructive spin wave interference. Second, we vary the strength of the bias magnetic field ±6 Oe and measure the change of the output inductive voltage in the vicinity of the destructive interference point.

**Figure 5:**
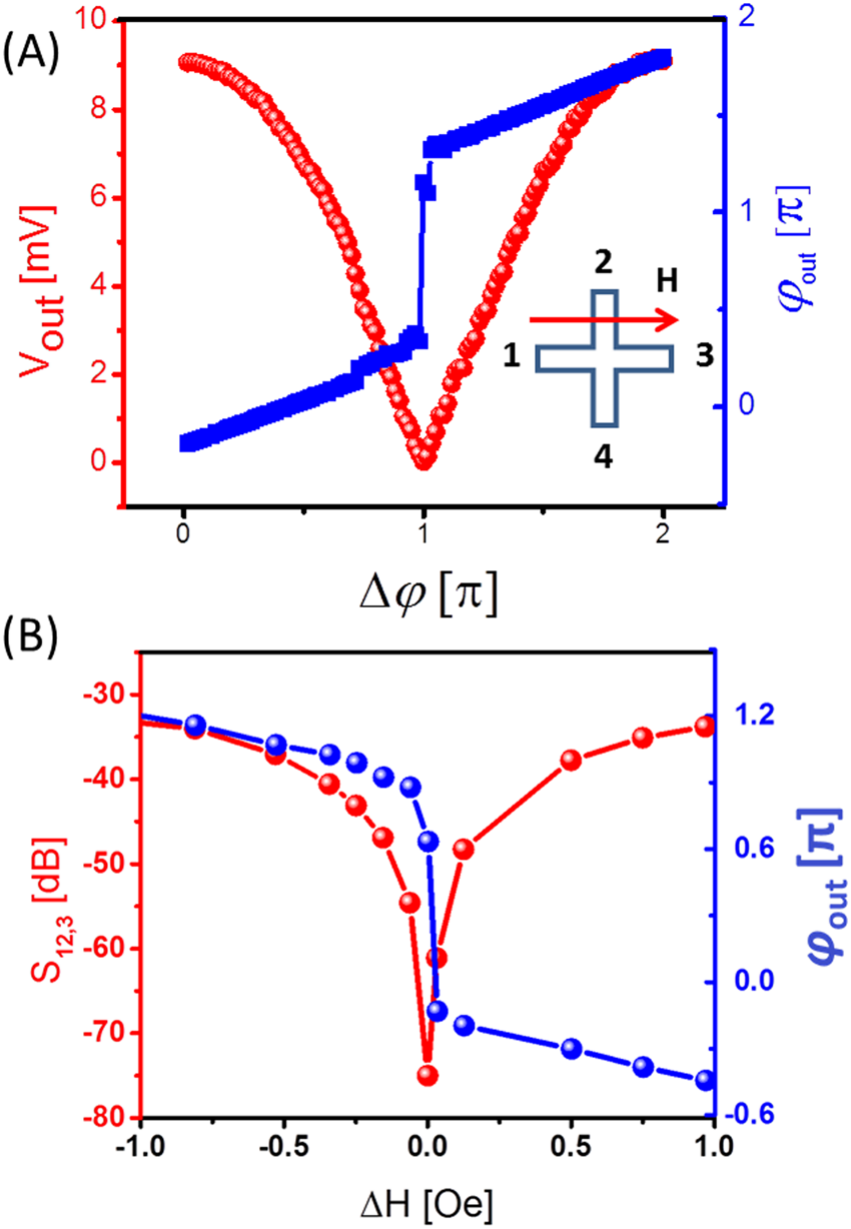
Experimental data obtained for magnetic field directed along the virtual line connected ports 1 and 3. (**A**) The amplitude (red markers) and the phase (blue markers) of the inductive voltage detected at port 3 as a function of the spin wave phase difference. Output voltage maxima correspond to the constructive spin wave interference. The minimum of the inductive voltage corresponds to the destructive spin wave interference. (**B**) Output voltage dependence on the magnetic field in the vicinity of the destructive interference point.

We carried out three sets of experiments aimed to show the change in the output inductive voltage with respect to changing magnetic field at different directions of the magnetic field. The operational frequency *f* is 4.95 GHz, and the bias magnetic field is *H* = 1074 Oe in all cases. The input microwave power at ports 1 and 2 is −6 dBm (0.25 mW). All experiments are performed at room temperature. In Fig. 5, we present experimental data for the bias magnetic field *H* directed parallel to the virtual line connecting ports 1 and 3 as illustrated in the inset. Figure 5(A) shows the amplitude (red markers) and the phase (blue markers) of the inductive voltage detected at port 3. On can clearly see the result of the spin wave interference, which provides the maximum output voltage of about 9 mV in the case of constructive spin wave interference (i.e. phase difference between the interfering spin waves Δ*φ*_12_ = 0*π*, 2*π*). The output has a minimum of about 10 μV in the case of the destructive spin wave interference (i.e., Δ*φ*_12_ = 1*π*). This is the most sensitive regime of operation according to the physical model described in the previous section. We fix the position of the phase shifters and attenuators to keep the sensing element in the destructive interference regime. As the next step, we vary the strength of the bias magnetic field. In Fig. 5(B), we present the experimental data showing the change in the output voltage as a function of the bias magnetic field within the destructive interference point. The data show a prominent signal variation of approximately 40 dB per 1 Oe.

**Figure 6:**
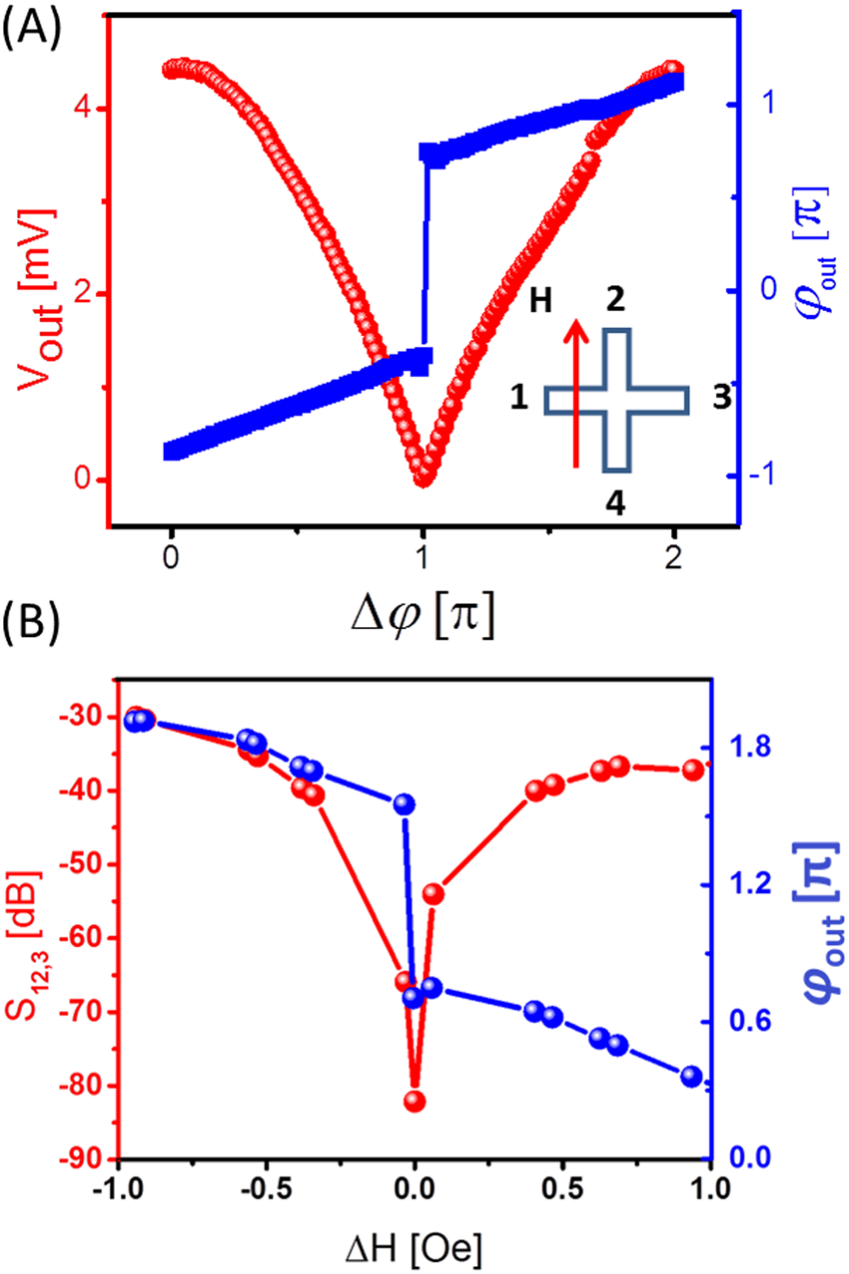
Experimental data obtained for magnetic field directed along the virtual line connected ports 2 and 4. (**A**) The amplitude (red markers) and the phase (blue markers) of the inductive voltage detected at port 3 as a function of the spin wave phase difference. (**B**) Output voltage dependence on the magnetic field in the vicinity of the destructive interference point.

**Figure 7:**
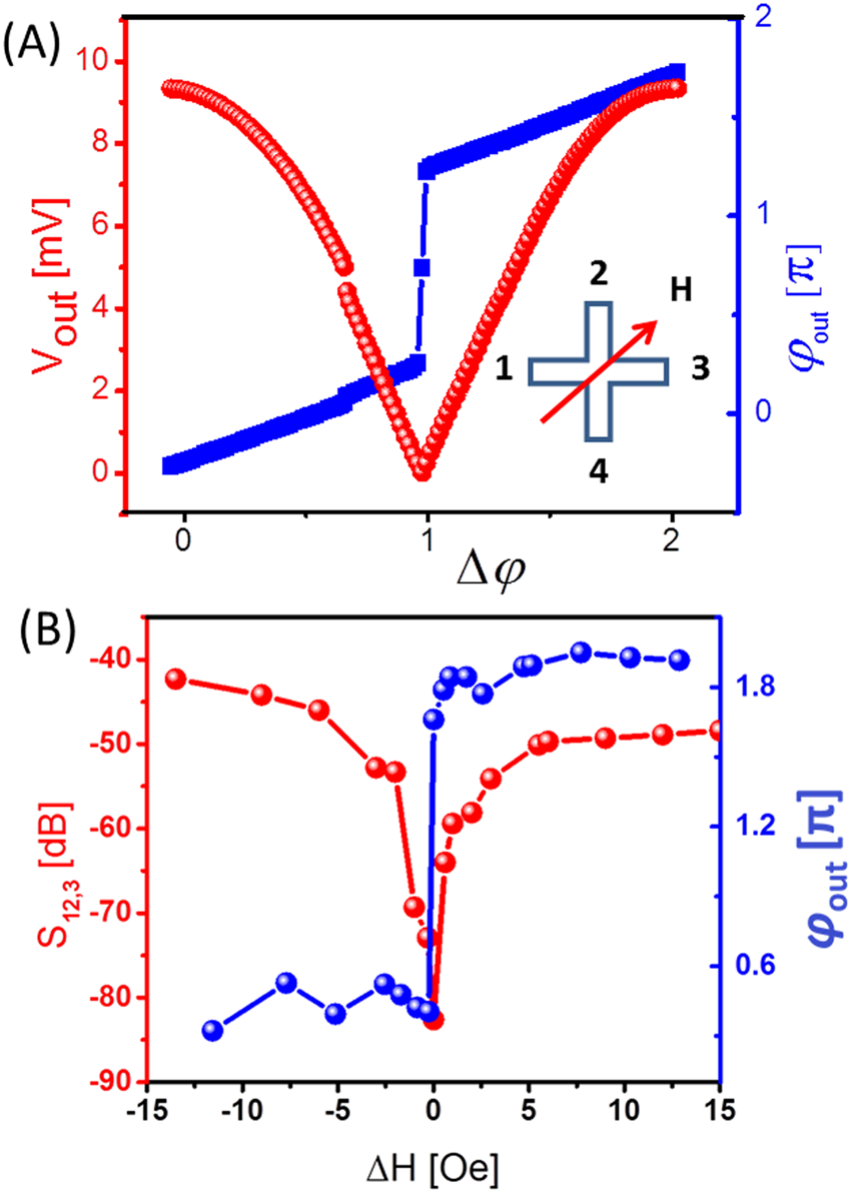
Experimental data obtained for magnetic field directed at 45^0^ to the virtual line connected ports 1 and 3. (**A**) The amplitude (red markers) and the phase (blue markers) of the inductive voltage detected at port 3 as a function of the spin wave phase difference. (**B**) Output voltage dependence on the magnetic field in the vicinity of the destructive interference point.

Similar experiments have been performed for two different orientations of the bias magnetic field. Figure 6(A)shows the amplitude (red markers) and phase (blue markers) of the inductive voltage detected at port 3. The bias magnetic field is directed perpendicular to the virtual line connecting ports 1 and 3. The output voltage attains its maximum at about 4.2 mV, which corresponds to the constructive spin wave interference. The minimum voltage of about 1 μV is detected in the case of the destructive spin wave interference. The change in the output voltage due to the variation in the bias magnetic field in the vicinity of the destruction interference point is shown in Fig. 6(B). The amplitude of the output voltage increases drastically (i.e., more than 50 dB) with the 1 Oe change of the bias magnetic field. Finally, we repeated measurements for bias magnetic field directed at 45^0^ with respect to the virtual line connecting ports 1 and 3. The experimental data are presented in Fig. 7(A) and (B). The change in the output voltage within the destructive interference point is relatively small (i.e., 5 dB per 1 Oe) as compared to the previous two experiments. As expected, the 45^0^ configuration shows the lowest sensitivity to the bias magnetic field variation, as the phase difference among the interfering spin waves occurs only in the cross center. The accuracy of the inductive voltage measurements is ±0.00046 mV.

## Discussion

**Figure 8:**
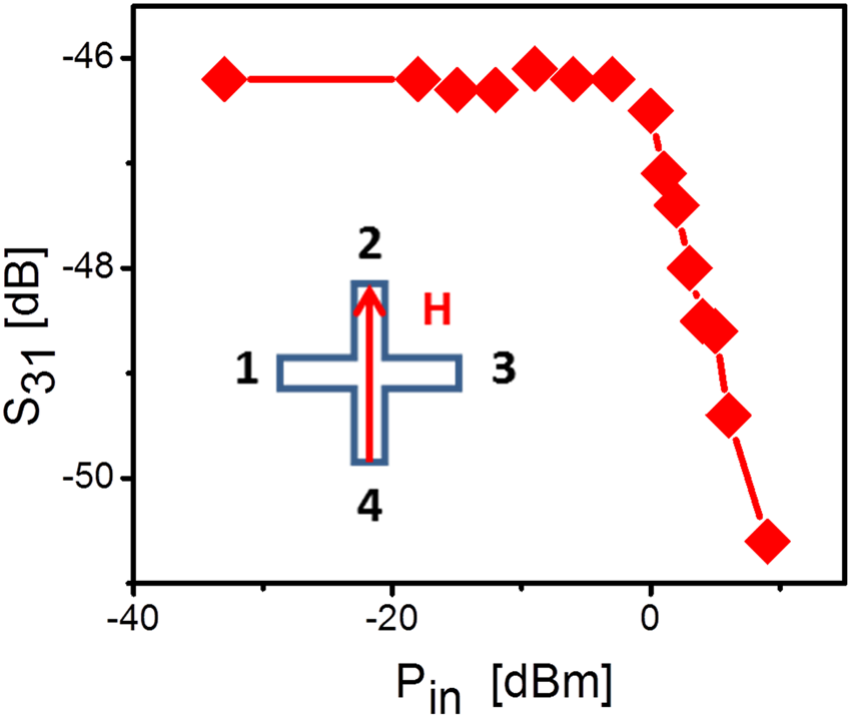
Experimental data showing the transmission characteristic as a function of input power. The transmission in YIG prototype drops due to the spin wave instability at the input power exceeding 0 dBm.

Experimental data presented above demonstrate a prominent change in the amplitude and phase of the output inductive voltage in the vicinity of the destructive spin wave interference point. In this section, we estimate the sensor sensitivity, and discuss potential advantages and limits of the proposed device. The transfer function ${S}_{B}^{V}$ (V/T) is the key sensor characteristic, which relates the input (magnetic field) to the output (inductive voltage). The maximum value of ~1 mV per 1 Oe was detected for the in-plane magnetic field directed along the virtual line connecting ports 1 and 3 as it is shown in Fig. 5. Note that the experimental data were obtained for the first, non-optimized prototype device. For instance, the input power for the spin wave generating antennas was only −6 dBm (0.25 mW). The transfer function can be further enhanced by applying a higher input power. The same YIG device can sustain operation at higher input power level of 0 dBm (1 mW). There are certain limits for pumping energy into the spin wave signals related to the dynamic instabilities^24^. In Fig. 8, we present experimental data showing the dependence of the transmitted signal on the input power. The instability restricts the input power of the YIG prototype at the level about +1 dBm. However, the relative change of the output inductive voltage exceeds 40 dB/1 Oe. The standard low noise electronics (e.g., an operational amplifier) allows one to amplify the output inductive voltage over the orders of magnitude^30^.

The second important characteristic is the intrinsic noise of the sensor, conventionally referred at the sensor’s input, in units of the square root of an equivalent magnetic power spectrum, *B*_*n*,eq_(*f*)^9^. Usually, the time variations of the output voltage are recorded, Fourier transformed, and then divided by the transfer function ${S}_{B}^{V}$ to get *B*_*n*,eq_(*f*), expressed in T/√Hz^9^. According to the formalism described^12^, the spectral density of the effective external magnetic field that describes thermal fluctuations in a medium with magnetic losses can be evaluated as the intrinsic thermal noise of magnetic material, which can be estimated as follows:

11 $${B}_{n}(f)=\sqrt{\frac{T{\rm{\Delta }}H}{4\pi V\gamma {M}_{S}^{2}}}.$$

Here *T* is the absolute temperature in erg and *V* is the magnetic film volume. The estimates have been obtained for a YIG sample with the following parameters Δ*H* = 1 Oe, *M* = 140 Oe, *V* = 2.5 × 10^−4^ cm^3^, frequency band 1 Hz, show 10^−15^ *T*/√*Hz* at room temperature^12^. Note that the output of the spin wave-based magnetometer is an AC inductive voltage. The latter allows one to exploit the phase lock-in amplifier technique^31^. Taking into account the RF operating range and well-defined output frequency, signals up to 1 million times smaller than the noise level can be detected. Altogether, it makes feasible to reach the detection level of cooled SQUIDs (i.e., attotesla) but with YIG sensors operating at room temperature.

Detecting the output phase provides us an alternative way for the magnetic field sensing. The phase of the output exhibits an 180^0^ abrupt jump near the destructive interference point. The jump occurs with less than 1 Oe variation of the bias magnetic field. Thus, the change in the magnetic field is related to the change in the output phase. In this scenario, the maximum field sensitivity is defined by the precision of the phase measurement. A sub-micro-degree phase measurement technique was demonstrated for the lock-in amplifiers^32^. The ultimate sensitivity of the interferometer combined with a phase lock-in amplifier may exceed 10^−13^ Tesla. There are several advantages of using the phase measurements compared to the amplitude-based approach. The phase change does not depend on the amplitudes of the input signals, which allows one to minimize the power consumption. The sign of the phase change is directly related to the decrease (increase) in the magnetic field while the amplitude of the output is almost symmetric (e.g., as shown in Figs 5, 6 and 7). A combination of the amplitude and phase measurements makes it possible to detect a change in the amplitude and direction of the sensing magnetic field.

An extremely fast data accumulation is another appealing property of the proposed magnetometer. The time delay is limited by two factors: the spin wave propagation time, and output voltage averaging time. The spin wave propagation time in the millimeter-long prototype device is about 0.1 µs. It takes at least one period of oscillations (0.1 ns) to detect an average amplitude of the output signal. There are pros and cons associated with downscaling of the size of the sensing element. On one hand, downscaling favors the proposed magnetometer by reducing its time delay, increasing operation frequency, and minimizing the thermal noise. On the other hand, shortening of the length of the arms reduces the phase difference between the spin waves propagating through the orthogonal arms (see eq. 7). Any shortening of the arm length should be accompanied by the reduction in the thickness *d* in order to preserve the phase sensitivity (Δ*φ*/*δH* ~ *l*/*d*). It should be noted that the formalism presented in this work (i.e., eq. (7)) is valid for the magnetostatic spin waves where the dipole-dipole interaction is dominant^26^.

Potentially, it can be possible to build a sub-micrometer size interferometer operating on the exchange spin waves. Such a device can have some superior properties stemming from minimization of the propagation losses and reduction of the propagation time^33^. For instance, the time delay in a 500 nm structure with a velocity of exchange magnons of 10^3^ m/s is only 0.5 ns^33^. The frequency of the exchange magnons achieves 7 THz at the edge of the first Brillouin zone^34^. At the same time, exchange waves may be less sensitive to the external magnetic field variation as compared to the dipole-dipole spin waves due to the strong exchange coupling.

The sensing elements made of YIG or similar ferrite materials possess a wide temperature operating range, from cryogenic to the 560 K. One more appealing property of the proposed magnetometer is that a large number of interferometers can be combined in a one detector network. It is possible to build a network comprising a number of interferometers in a phased array in which the relative phases (amplitudes) of input spin waves are adjusted in such a way that the incoming magnetic signals of interest are amplified while other signal, coming from undesired directions, are suppressed. The phase arrays are currently used in a variety of applications. For example, the MESSENGER spacecraft (mission to the planet Mercury arrived 18 March 2011) was the first deep-space mission with a phased-array antenna for communications^35^. Potentially, the spin wave interferometers can be arranged in a magnetic telescope for the outer space exploration.

The need for the bias magnetic field is the main technological disadvantage of the described magnetometer. The most sensitive regime of operation requires an accurate adjustment of the bias magnetic field and the operational frequency to ensure the destructive spin wave interference at the one of the outputs. This issue can be resolved by implementing magnetic materials with the out-of-plane magnetization and/or utilization of antiferromagnetic materials. A lack of experimental data on spin wave interference in such materials prevents a qualitative analysis. The major magnetometer characteristics such as time delay, signal bandwidth, magnetic field sensing range are directly related to the spin wave dispersion and attenuation, which define the physical limits of using spin waves for the magnetic field sensing. There is a tradeoff between material parameters (i.e. *M*_*s*_, *γ*), element geometry (i.e. *l/d* ratio) and the operating range (i.e. *f*, *H*). For example, the phase sensitivity can be enhanced by increasing the *l/d* ratio. However, the increase in the propagation length *l* is associated with an additional signal damping and degradation of the transfer characteristics.

Improving the device noise characteristics is the major task for further magnetometer development. In our preceding work^16^, we presented the results of 15,000 subsequent measurements on the variation of the output characteristics for the constructive and destructive interference. The normalized noise spectral density S_V_/V^2^ was estimated on the order of 10^−11^ 1/Hz, which was mainly dominated by the electrical noise. Achieving the ultimate limit of 10^−15^ *T*/√*Hz* in YIG at room temperature will require a special study. In this work, we used only one antenna for the output voltage detection. Utilization of a second output will allow one to take an advantage of a differential amplifier, which has been proven as an efficient tool for noise suppression in the low-voltage systems^36, 37^.

To conclude, we conducted proof-of-concept measurements to demonstrate a magnetometer based on spin wave interference, and described advantages of the proposed device. There is a variety of methods for improvements of the sensing element design. The geometry of the cross junction can be optimized to ensure a wider overlap among the MSSW and BVMSW signals. The integration of spin wave interferometers in a network is one of the promising directions for further research. We argue that our room-temperature spin wave magnetometers can compete with SQUIDs in sensitivity. Other advantages include compactness, fast data acquisition, and a wide temperature operating range.

## Methods

### Device Fabrication

The sensing element is a cross junction made of single crystal Y_3_Fe_2_(FeO_4_)_3_ film. The film was grown on top of a (111) Gadolinium Gallium Garnett (Gd_3_Ga_5_O_12_) substrate using the liquid-phase epitaxy technique. The micro-patterning was performed by laser ablation using a pulsed infrared laser (λ ≈ 1.03 μm), with a pulse duration of ~256 ns. The YIG cross has the following dimension: the length of the each waveguide is 3.65 mm; the width is 650 µm; and the YIG film thickness is 3.8 µm; and saturation magnetization of 4*πM*_0_ ≈ 1750 *Oe*. There are four Π-shaped micro-antennas fabricated on the edges of the cross. Antennas were fabricated from a gold wire of thickness 24.5 µm and placed directly at the top of the YIG surface.

### Measurements

The antennas are connected to a programmable network analyzer (PNA) Keysight N5241A. Two of the antennas are used to generate two input spin waves. The inductive voltage is detected by the other two antennas. The set of attenuators (PE7087) and phase shifters (ARRA 9428A) is used to control the amplitudes and the phases of the interfering spin waves.

## References

[1] Shah VK, Wakai RT (2013). A compact, high performance atomic magnetometer for biomedical applications. Physics in Medicine and Biology.

[2] Tao, W., Gao, J., Zhang, F., Cai, W. & Deng, P. *High-Tc squid sensors and its application in bio-magnetic measurement* (2012).

[3] Begus S, Fefer D (2007). An absorption-type proton NMR magnetometer for measuring low magnetic fields. Measurement Science & Technology.

[4] Nikiel, A. *et al*. Ultrasensitive He-3 magnetometer for measurements of high magnetic fields. *European Physical Journal D* **68**, doi:10.1140/epjd/e2014-50401-3 (2014).

[5] Ripka, P. *Magnetic sensors and magnetometers - Preface* (2001).

[6] Day, G. W. In *Optical Fiber Sensors*. *Proceedings of the 6th International Conference*. *OFS ‘89*. *S*pringer-V*erlag*. 1989, *pp*. *250-*4. Berlin, West Germany. (eds H. J., Arditty, J. P., Dakin & R. T., Kersten).

[7] Okamura H (1990). Fiber-optic magnetic sensor utilizing the Lorentzian force. Journal of Lightwave Technology.

[8] Koo KP, Bucholtz F, Dagenais DM, Dandridge A (1989). A compact fiber-optic magnetometer employing an amorphous metal wire transducer. IEEE Photonics Technology Letters.

[9] Robbes D (2006). Highly sensitive magnetometers - a review. Sensors and Actuators a-Physical.

[10] Drung D (2007). Highly sensitive and easy-to-use SQUID sensors. Ieee Transactions on Applied Superconductivity.

[11] Vetoshko PM, Volkovoy VB, Zalogin VN, Toporov AY (1991). Measuring low alternating magnetic-fields by means of Bi-Containing rare-earth ferrite-garnet films with planar anisotropy. Journal of Applied Physics.

[12] Vetoshko PM, Valeiko MV, Nikitin PI (2003). Epitaxial yttrium iron garnet film as an active medium of an even-harmonic magnetic field transducer. Sensors and Actuators a-Physical.

[13] Gertz F, Kozhevnikov A, Filimonov Y, Khitun A (2015). Magnonic Holographic Memory. Magnetics, IEEE Transactions on.

[14] Gertz, F., Kozhevnikov, A., Filimonov, Y., Nikonov, D. E. & Khitun, A. Magnonic Holographic Memory: From Proposal to Device. *IEEE Journal on Exploratory Solid-State Computational Devices and Circuit*, doi:10.1109/JXCDC.2015.2461618 (2015).

[15] Kozhevnikov A, Gertz F, Dudko G, Filimonov Y, Khitun A (2015). Pattern recognition with magnonic holographic memory device. Applied Physics Letters.

[16] Balynsky, M. *et al*. Magnonic interferometric switch for multi-valued logic circuits. *Journal of Applied Physics* **121**, doi:10.1063/1.4973115 (2017).

[17] Davies, C. S. *et al*. Towards graded-index magnonics: Steering spin waves in magnonic networks (vol. 92, 020408, 2015). *PHYSICAL REVIEW B* **95**, doi:10.1103/PhysRevB.95.019901 (2017).

[18] Sadovnikov, A. V. *et al*. Magnonic beam splitter: The building block of parallel magnonic circuitry. *Applied Physics Letters* **106**, doi:10.1063/1.4921206 (2015).

[19] Silva TJ, Lee CS, Crawford TM, Rogers CT (1999). Inductive measurement of ultrafast magnetization dynamics in thin-film Permalloy. Journal of Applied Physics.

[20] Covington, M., Crawford, T. M. & Parker, G. J. Time-resolved measurement of propagating spin waves in ferromagnetic thin films. *Physical Review Letters* **92**, doi:10.1103/PhysRevLett.92.089903 (2004). 10.1103/PhysRevLett.89.237202 12485035

[21] Bailleul M, Olligs D, Fermon C (2003). Propagating spin wave spectroscopy in a permalloy film: a quantitative analysis. Applied Physics Letters.

[22] Kittel, C. Introduction to Solid State Physics, 8th Edition (2005).

[23] Damon RW, Eshbach JR (1961). Magnetostatic modes of a ferromagnet slab. Journal of Physics and Chemistry of Solids.

[24] Gurevich, A. G. & Melkov, G. A. (CRC Press Inc., New York, 1996).

[25] Eschbach, J. & Damon, R. *J*. *Phys*. *Chem*. *Solids* **19**, 308 (1961).

[26] Kostylev, M. P., Serga, A. A., Schneider, T., Leven, B. & Hillebrands, B. Spin-wave logical gates. *Applied Physics Letters* **87**, 153501-153501-153503 (2005).

[27] Demokritov, S. O. *et al*. Tunneling of dipolar spin waves through a region of inhomogeneous magnetic field. *Physical Review Letters* **93**, doi:10.1103/PhysRevLett.93.047201 (2004). 10.1103/PhysRevLett.93.047201 15323787

[28] Donahue, M. J. & Porter, D. G. OOMMF User’s Guide, Version 1.0 Interagency Report NISTIR 6376 (National Institute of Standards and Technology, Gaithersburg, MD, 1999).

[29] Covington, M., Crawford, T. M. & Parker, G. J. Time-resolved measurement of propagating spin waves in ferromagnetic thin films. *Physical Review Letters* **89**, 237202-237201-237204 (2002). 10.1103/PhysRevLett.89.237202 12485035

[30] Huijsing, J. *Low Noise and Low Offset Operational and Instrumentation Amplifiers* (2011).

[31] Schauber MJ, Newman SA, Goodman LR, Suzuki IS, Suzuki M (2008). Measurement of mutual inductance from the frequency dependence of impedance of AC coupled circuits using a digital dual-phase lock-in amplifier. American Journal of Physics.

[32] Walker, W. D. & Ieee. *Sub-microdegree Phase Measurement Technique using Lock-in Amplifiers* (2008).

[33] Chumak, A. V., Serga, A. A. & Hillebrands, B. Magnon transistor for all-magnon data processing. *Nature Communications* **5**, doi:10.1038/ncomms5700 (2014). 10.1038/ncomms5700 PMC4143911 25144479

[34] Plant JS (1977). Spin-wave dispersion curves for yttrium iron-garnet. Journal of Physics C-Solid State Physics.

[35] Wallis, R. E. & Sheng, C. *Phased-array antenna system for the MESSENGER deep space mission*. (2001).

[36] Tsarapkin, D. P. & Ieee. in 2005 *Ieee International Frequency Control Symposium and Exhibition IEEE International Frequency Control Symposium* 534–538 (2005).

[37] Ivanov EN, Tobar ME, Woode RA (1998). Applications of interferometric signal processing to phase-noise reduction in microwave oscillators. Ieee Transactions on Microwave Theory and Techniques.

